# CO_2_ Concentrations and Thermal Comfort Analysis at Onsite and Online Educational Environments

**DOI:** 10.3390/ijerph192316039

**Published:** 2022-11-30

**Authors:** Alba Alegría-Sala, Elisenda Clèries Tardío, Lluc Canals Casals, Marcel Macarulla, Jaume Salom

**Affiliations:** 1 Enginyeria del Medi Ambient (ENMA), Department of Project and Construction Engineering (DPCE), Universitat Politècnica de Catalunya (UPC), 08028 Barcelona, Spain; 2 Thermal Energy and Building Performance Group, Catalonia Institute for Energy Research (IREC), 08930 Catalonia, Spain; 3 Group of Construction Research and Innovation (GRIC), Department of Project and Construction Engineering (DPCE), Universitat Politècnica de Catalunya (UPC), 08222 Barcelona, Spain

**Keywords:** IAQ, thermal comfort, on-site education, online education, COVID-19

## Abstract

In building areas with high occupancy, such as classrooms, transmission routes of SARS-CoV-2 are increased when indoor air quality is deficient. Under this scenario, universities have adopted ventilation measures to mitigate contagious environments. However, the lack of adequate equipment or designs in old educational buildings is a barrier to reach minimum requirements. This study aims to quantify the indoor air quality and thermal comfort at universities and compare it to conditions in students’ households. In this regard, several classrooms in buildings of the Polytechnic University of Catalonia were monitored for temperature, CO_2_ concentration and relative humidity. The people who used these classrooms were surveyed about their comfort perceptions. A sample of students was also monitored at their homes where they reported to studying during the exam period. By means of point-in-time surveys, students reported their daily comfort, for comparison with the monitored data. The results show that the recommendations for CO_2_ concentration, temperature, and relative humidity are not always met in any of the study spaces. These factors are more critical at universities due to the high occupancy. In addition, the surveys highlighted the perception that the environment is better at home than at university.

## 1. Introduction

Since the beginning of the COVID-19 pandemic, declared on 11 March 2020 by the World Health Organization (WHO) [1], worldwide governments have implemented several measures to try to minimize the rapid proliferation of the virus. SARS-CoV-2 is mainly spread through the respiratory tract and close contact, and droplet transmission is the main mode of infection [2]. Based on this knowledge, experts recommended keeping a minimum distance of 1 m between people, ceasing indoor activities, and, when they could not be suspended, controlling the number of participants and implementing personal protective measures, such as wearing face masks and practicing strict hand disinfection [3].

In the first stages of the pandemic, health centers were at full capacity. Consequently, many countries enforced a strict lockdown and imposed the above recommendations for cases when interaction was unavoidable. In Spain, as in many other European countries, education was moved entirely online or was suspended during this period. It was not until many months later that a gradual return to a face-to-face model was possible. To ensure a safe return to shared indoor spaces, the maximum seating capacity was reduced to 70% in the case of the Universitat Politècnica de Catalunya (UPC) [4], and ventilation became a priority, since it has been proven an effective tool in reducing the incidence of infections [5,6].

To determine ventilation efficiency, the standards provide different measurement methods depending on the air pollutants that are analyzed. As stated by international organizations such as the American Society of Heating, Refrigerating and Air-Conditioning Engineers (ASHRAE) or the World Health Organization (WHO) [7], the most common air pollutants that contribute to poor indoor air quality (IAQ) are carbon dioxide (CO_2_), carbon monoxide (CO), formaldehyde (HCHO), nitrogen dioxide (NO_2_), sulfur dioxide (SO_2_), volatile organic compounds (VOCs), and particulate matter (PM2.5 and PM10). Given that CO_2_ is strongly linked to human bioeffluents [8], it offers data on occupant densities and can be used to predict indoor SARS-CoV-2 concentrations [9].

This is why governments have been pointing to certain concentrations levels of CO_2_ to minimize the risk of contagion in closed spaces. In the case of Spanish universities, the maximum recommended value was set at 800–1000 ppm [10,11,12]. However, these values are based on standards developed before the global pandemic outbreak. Several studies have noted that lower limits should be maintained in such cases. According to a study by Antonio López et al. [13], levels should not rise above 700 ppm as this indicates that 1% of the air breathed by a person has already been inhaled by another occupant. In contrast, Alessia Di Gilio et al. [14] present four risk levels associated with CO_2_ concentration: “low risk” when CO_2_ levels remain below 700 ppm, “moderate risk” for concentrations between 700 and 800 ppm, “high risk” for concentrations in the range of 800 to 1000 ppm, and “very high risk” when concentrations exceed 1000 ppm. Note that these levels estimate an outdoor CO_2_ concentration of 400 ppm and that the concentrations are not intended for residential buildings, as the risk of infection in these places is not as significant. However, given that indoor air quality is important to avoid contagion and as a tool to reduce the impact that pollutants can have on health, there are also regulations on the levels of CO_2_ that must be maintained in dwellings, as is the case of the ISO EN16798–1–2019 regulation [15].

Other parameters such as temperature and relative humidity have proven to be important in dealing with the spread of COVID-19 viruses. Similar responses have been found to those observed with influenza-type viruses [16]. The survival of influenza infectious agents is better in environments with low temperatures, which facilitate their propagation [17]. As Lowen et al. conclude [18], the optimum temperature to control propagation is 30 °C, which would lead to inadequate thermal comfort. At lower temperatures, its spread becomes dependent on the relative humidity. For the purpose of achieving thermally comfortable but safe environments, they indicate that the combination to be achieved is a temperature of 20 °C or higher and a relative humidity of 50% or above [19]. Note that temperatures lower than 16 °C have are hazardous to the occupants’ health [20]. High temperatures can also have a negative impact on humans, and, even if it has been difficult to identify an adequate threshold, a maximum indoor temperature of 26 °C may be the most suitable for ensuring a safe environment [21].

Notably, the recommendations and guidance given to prevent COVID-19 from spreading have been defined regardless of the type of ventilation available in buildings. It has been proven that in naturally ventilated spaces the indoor environmental conditions are deeply affected by the outdoor weather conditions and the occupants’ behavior [22].

Apart from the negative impacts on health, high concentrations of CO_2_ levels present a drop in the speed of cognitive functions [23,24]. Furthermore, the perception of workload for a particular job is substantially greater at 17 °C and 28 °C than at 21 °C, and both accuracy and speed drop with the rise in total workload [25]. Additionally, Cui et al. indicate that there is no change in performance between 22 °C and 26 °C, whereas the ideal temperature for motivation, understood as the willingness to work, is set at 24.7 °C [26].

Nonetheless, today, the level of concern about COVID-19 has been reduced, and it is predicted that the pandemic could be terminated by 2022 [27]. However, the virus could evolved towards higher infectivity by developing greater resistance to antibodies [28], so future outbreaks of the disease cannot be discarded [27]. Additionally, climate change has been found to worsen numerous infectious illnesses [29], potentially leading to new health crises.

Given the current situation and considering that future health emergencies could eventually result in new housebound situations [27], it has been considered important to determine the suitability of learning spaces at universities and in students’ homes. Therefore, an environmental assessment is carried out by means of CO_2_ concentrations, temperature, and relative humidity, as well as by studying the thermal comfort of those spaces, which have been calculated using the adaptive model presented in the ISO EN16798–1–2019 [15] and through surveys. The study also assesses the maximum capacity set by the university to ensure a safe return to on-site education. The results are supplemented with the overall perception of students about their environmental comfort when learning from home or at the university. This is done by evaluating five main parameters: air quality, noise, lighting, and thermal comfort in winter and in summer.

## 2. Materials and Methods

**Figure 1:**
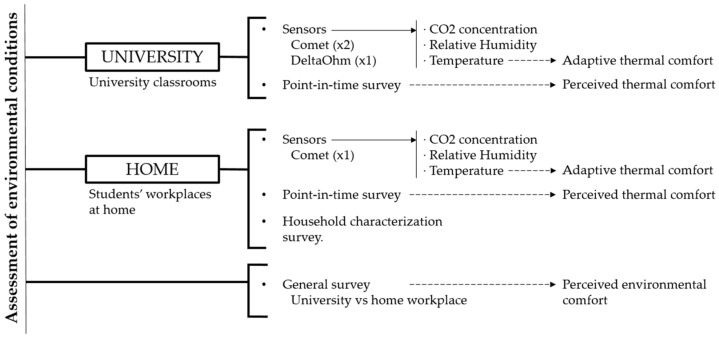
Data collection methodology framework.

The current study is based on data collected through two monitoring campaigns carried out during the 2021–2022 academic year (see Figure 1). The first campaign aimed to gather information on the environment quality in university classrooms; therefore, a total of 23 measurements were carried out in classrooms at 3 different university buildings: Escola Superior d’Enginyeries Industrial, Aeroespacial i Audiovisual de Terrassa (ESEIAAT), Facultat de Nàutica de Barcelona (FNB), and Escola Tècnica Superior d’Enginyeria Industrial de Barcelona (ETSEIB). The collected data was processed considering winter and summer seasons separately. The first dataset corresponds to measurements carried out from November to April and the second set to those completed between May and October.

The second monitoring campaign counts with 17 measures performed at students’ workspaces at home. The volunteers in this second monitoring campaign were recruited at the ETSEIB University, and the requested condition was that they studied from home during the exam periods, which in this case went from Saturday 2 April to Friday 8 April 2022 for the mid-term exams, corresponding to winter season, and from Saturday 28 May to Sunday 19 June 2022 for the final exams period, covering the summer season analysis. Note that these timespans are not lockdown periods during which online education is provided, as currently only the on-site model is applied at the UPC. However, during exams periods, no lectures are held and some students spend their studying time at home, offering a good representation of what could happen in times of online education.

Environmental quality was evaluated through CO_2_ concentration, air temperature, and relative humidity. It should be noted that although knowing that there are other types of pollutants to define IAQ, this study focusses on CO_2_ levels as it is strongly related to bioeffluents, and, therefore, CO_2_ is a useful index to determine the risk of contagions in closed spaces.

In the universities, these parameters were monitored using three IAQ dataloggers: two units of the Comet U3430 (Comet) model and one of the DeltaOHM HD32.3TC (DeltaOHM) sensor. However, only one Comet U3430 sensor was provided to students for the study in dwellings due to the availability of such a number of sensors and for practical reasons; Comet U3420 sensors are smaller and have a longer battery duration, thus they are easier to transport and place in the study spaces. The technical data for each sensor is shown in Table 1.

The recording interval set for classrooms’ measurements was of one minute and the studied interval was equal to the duration of the lessons. The sensors were placed on separate tables at a height between 0.9 m to 1.2 m, depending on the sensor support and the table height of the classroom, which corresponds to the breathing zone [30]. Moreover, the location of sensors was set to avoid disturbing students and the regular course of lessons. For the present study, the value of each parameter corresponds to the average of the readings taken by each of the sensors. Under no circumstances were the typical classroom conditions altered. No adjustments to the ventilation or climatization were made, and no requirements were established for the clothing or behavior of the occupants.

For the analysis of work spaces at home, volunteers were given a set of instructions to ensure accurate readings of the sensors in a similar distribution to those at university. The guidelines given for the correct placement of the sensor were (i) to place the Comet U3430 in the study room, (ii) to not move the sensors once they have been placed, (iii) to keep them away from direct sunlight or lamp radiation, and (iv) to place them away from heat sources such as radiators or electronic equipment. No proof of the correct location of the sensors was requested for privacy reasons; however, the data was examined to detect anomalies. For these measurements, a recording interval of two minutes was established due to the memory capacity of the sensors.

Recorded data from each measurement were evaluated by means of boxplots produced with Python data visualization Seaborn library [31]. Atypical values were detected in no cases, and the upper and lower extremes were found to be consistent. However, as further explained in the results section, house measurements are filtered between 00:00 h and 6:58 h since the increase in CO_2_ concentration during night is higher than during the rest of the day, which is interpreted as an effect caused by sleeping hours when spaces are normally less ventilated.

The indoor environmental quality was evaluated through different limits. To analyze the CO_2_ concentration in universities, the risk levels proposed by Alessia Di Gilio et al. [14] were used while for the analysis of dwellings the limits presented in the ISO EN16798–1–2019 [15] regulation were applied. According to this regulation, the intended comfort range for homes is IEQ_II_ for new construction buildings and IEQ_III_ for existing ones. The IEQ_IV_ category should be avoided and is only tolerable for brief intervals.

Considering an outdoor CO_2_ of 416 ppm, which is the 2021 annual mean given by the ESRL’s Global Monitoring Laboratory (GML) of the National Oceanic and Atmospheric Administration (NOAA) in Mauna Loa [32], the abovementioned thresholds were adjusted, which led to Table 2 values.

Additionally, to validate the 70% of maximum capacity established institutionally in the event of a pandemic scenarios [4], the increase in CO_2_ concentration was assessed in relation to the level of occupancy of the classrooms. In this regard, all intervals in which classrooms were not ventilated were identified and organized based on the available volume per occupant.

The assessment of temperature and relative humidity was carried out using the ranges in RD 486/1997 [33], which establish the minimum health and safety requirements in workplaces where sedentary activities are performed. As stated in the document, the acceptable temperature range is between 17 °C and 27 °C, and the relative humidity should be between 30% and 70%. Nevertheless, since these limits do not contemplate pandemic situations, temperature and relative humidity thresholds of 20 °C and 50%, respectively, were also set, as they have been shown to be critical in the spread of influenza viruses. In this study, indoor environments with both parameters below these values were considered “undesirable environments” while the rest of the situations were classified as “desirable environments”.

In addition to the analysis of these parameters, environmental quality was also evaluated through the study of thermal comfort. The most commonly used thermal comfort indicator is the PMV index [34]. This model combines the theory of heat balance with the physiology of human thermoregulation and is based on laboratory studies carried out in climatic chambers [35]. However, in practice, environmental circumstances and human actions are much more dynamic, so the PMV model can under- or overestimate the comfort temperature, especially in buildings with passive ventilation strategies [36,37]. To solve this issue, De Dear and Brager developed the adaptive thermal comfort model based on field studies conducted around the world [38]. According to the adaptive model, users should be active participants in creating a balance between the human body and its surroundings. It also addresses the impact of climate, determining that the temperature of thermal neutrality tends to rise when the outside temperature rises.

The standards essentially introduce these two models and, although at the beginning the presence of the PMV model was imposed, consecutive updates led to widening the range of applicability of the adaptive model, recommending its use in naturally ventilated and mixed-mode ventilated environments [39]. Since most of the conducted measurements were held in naturally ventilated environments or, alternatively, in spaces with mixed ventilation, the mathematical model applied was the adaptive model, as described in ISO EN16798–1–2019 [15]. This model uses the operative temperature (*T_op_*, °C) as the main index, which relates indoor air temperature (*T_i_*) and radiant mean temperature (*T_rm_*) using Equation (1),

(1) $$T_{op}=A\cdot T_{i}\;\;+\;\;B\cdot T_{rm}$$

where *A* and *B* are parameters that are dependent on indoor air velocity (*v*_a_).

The parameters *v*_a_ and *T_rm_* were not recorded, since their measurement requires sensors that are more difficult to transport and could have disturbed the students in their households. Therefore, for the calculation, it was assumed that *T_rm_* was equal to *T_i_* and that *v*_a_ was lower than 0.2 m/s, which implies that *A* = *B* and consequently *T_op_* = *T_i_*.

Finally, this method correlates the *T_op_* index with the outdoor weather conditions using the outdoor running mean temperature (*T*_*o*,*rm*_) and the permissible temperature ranges shown in Table 3. *T*_*o*,*rm*_ is a weighted average of the temperature in the 7 days prior to monitoring as indicated in Equation (2),

(2) $$T_{o,rm}{}_{d}^{h}=\left(T_{o}{}_{d-1}^{h}\;+\;0.8\cdot T_{o}{}_{d-2}^{h}\;\;+\;\;\;0.6\cdot T_{o}{}_{d-3}^{h}\;\;+\;\;0.5\cdot T_{o}{}_{d-4}^{h}\;\;+\;\;0.4\cdot \;T_{o}{}_{d-5}^{h}\;\;+\;\;0.3\cdot T_{o}{}_{d-6}^{h}\;\;+\;\;0.2\cdot \;T_{o}{}_{d-7}^{h}\right)/3.8$$

where *d* corresponds to the day that is being considered, *h* is the hour considered to calculate the mean of the previous 24 h, and *T_o_* is the daily mean outdoor air temperature. The outdoor data was obtained using Meteocat API [40], which enables access to all the weather stations in Catalonia. The stations consulted for the comfort calculation in university classrooms were those closest to each engineering school, and, for the dwellings, the station located in Barcelona (El Raval) with code X4 was used.

In addition to the data collected through the sensors, volunteers were asked to complete different questionnaires (see Figure 1). All volunteers were told that participation was voluntary as was the decision to answer each of the questions. Participants were also asked whether they agreed with the privacy policy.

At universities, the occupants of the measured classrooms—students, teachers, and researchers—filled in a point-in-time survey that collected information on each person’s perceived thermal comfort, designed according to the ASHRAE 55–2017 standard [41]. To encourage participation, the survey was translated into English, Spanish, and Catalan, and a QR code was projected on the blackboard so everyone could scan it with their phones.

At home, the students who agreed to monitor their apartments answered two types of questionnaires: the environmental point-in-time comfort survey, answered at least once a day for gathering their thermal comfort perception, and the household characterization survey, answered only once.

Household characterization surveys collect detailed data about the household, its inhabitants, and their behavior. Some examples of the information gathered from the 41 questions are the household surface area, location, and equipment; the age, gender, and health of the inhabitants; and the use of equipment and ventilation. In addition, some questions focused on general perception levels (thermal, humidity, air quality, noise, and light) were asked. In all cases, the scale was from 1 to 7.

Finally, a general survey was developed to determine students’ overall perception of environmental quality when they were learning from home and at university. It was distributed to over 200 people, out of whom 43 responded. This 17-question survey asked about the engineering school building where students study and whether they had a specific space for working at home. They were asked to rate on a scale of 1 (discomfort) to 7 (comfort) the lighting, noise, air quality, and thermal comfort in summer and winter at the university and at home. They were asked to indicate the reasons for thermal discomfort in both spaces and the origin of other reasons for discomfort. Finally, the last question asked them to indicate in which of the two spaces they felt more comfortable.

## 3. Results

Three university buildings in the province of Barcelona were studied: the ESEIAAT TR1 building that was built in 1901 and has a living area of 12,802 m^2^; the FNB building that was constructed in 1932 and has a usable area of 3544 m^2^; and the engineering school, ETSEIB, the newest building as it was constructed 1964. It has a usable surface of 21,135 m^2^.

Of the measurements carried out in university classrooms, 81% corresponded to spaces where only natural ventilation is available. None of these classrooms have a cooling system. A total of 62% of the classrooms have centralized air heating, which does not allow for individual regulation of the spaces. The rest are equipped with water radiators. The heating is set to switch on and off based on the outside temperature, which is measured by means of a 1.5 m NTC sensor, and the time of day. During the night, from 20.30 h to 6.30 h, the system is switched off. From this time in the morning until 9.30 h, the heating starts if the recorded temperature is below 16 °C. Between 9.30 h and 17.00 h, the set point temperature is 14 °C. Finally, until the end of the day, the system works if the outside temperature is below 15.5 °C. In all cases, if the outside temperature exceeds the set point temperature by 1 degree, the system switches off automatically.

For classrooms with forced ventilation, the air quality is measured with CO_2_ sensors installed inside the rooms, and the system starts when readings reach 750 ppm. In these spaces, air conditioning is mainly provided by ceiling splits that, although they can be activated by the occupants, only work when the classroom temperature is not between 21 °C and 26 °C. This is measured by employing a sensor in the air conditioning equipment.

Regarding households, seven measurements were made in the winter and ten in the summer. Counting both seasons, a total of 13 students participated while 11 participants answered the characterization survey.

Through the characterization survey, it was found that respondents’ households were in Barcelona and surroundings (less than 25 km from the center) except for one household in Centelles, 50 km from Barcelona. The construction year of the houses are 1900, 1910, 1920, 1936, 1963 (two houses), 1966, 1978, 1988, 2005, and 2009. All the households with a cooling/heating system reported using it when people were at home and with local spatial and temporary use. Seven households had water radiators powered by natural gas, two had electric radiators, one had a heat pump, and one had no heating system. Only five households had a cooling system, and, of the other six, only three had fans. Participants reported using the cooling system or mechanical ventilation when the thermal perception was very hot and only in the occupied rooms. As for natural ventilation, only one household reported not opening the windows during the winter and another reported not opening them in the summer. The rest of the users aired their homes on varied schedules. However, only two reported airing the home at night and only during the summer. The respondents declared an acceptable perception for natural illumination, noise, and air quality in their homes. They evaluated it between 4.1 and 5.3 in winter and between 4.8 and 6.2 in summer on a scale from 1 to 7 points, where 1 is not acceptable.

**Figure 2:**
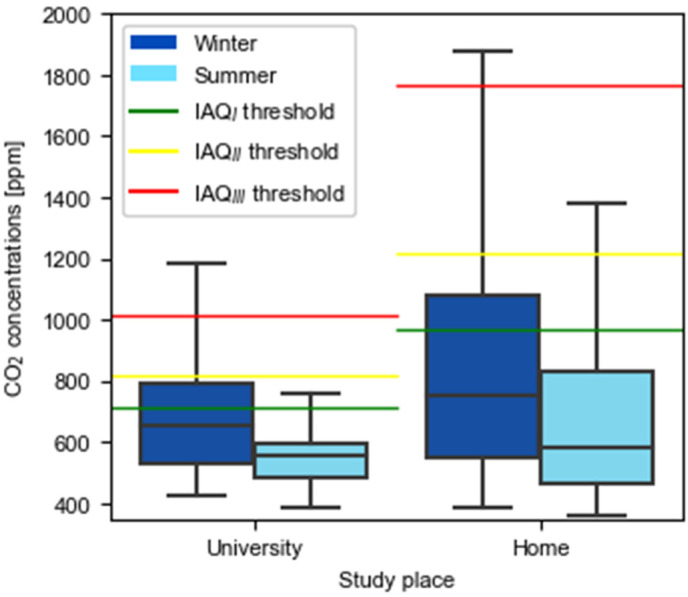
Recorded CO_2_ concentrations in universities and homes during winter and summer seasons.

As shown in Figure 2, CO_2_ levels were higher during the winter in both universities and homes. This is consistent since during the summer, windows are more easily opened as the outdoor temperature facilitates this, while in winter thermal comfort seems to be prioritized over ventilation. In general, universities have lower CO_2_ concentrations than homes, but IAQ thresholds vary because SARS-CoV-2 transmission is not considered a relevant indicator in households due to the low occupancy rates.

Focusing on universities, even if the median remains below 700 ppm, the results obtained indicate that safe conditions do not always prevail, since during the winter, 21% of the time the indoor air quality is outside of acceptable ranges. During the summer, concentrations above the recommended levels were also identified 7% of the time (Table 4).

**Figure 3:**
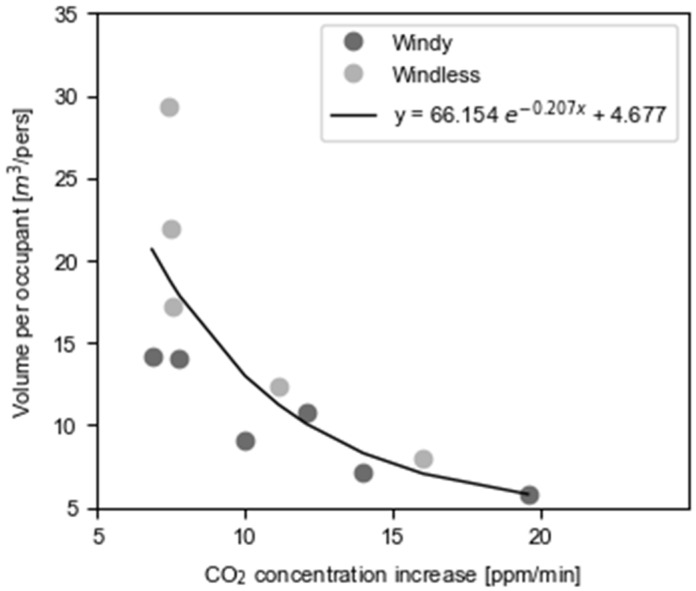
Increase in CO_2_ concentrations in non-ventilated spaces as a function of the available volume per occupant.

The analysis of the periods without ventilation shows that CO_2_ concentration increases exponentially as a function of occupancy through the polynomial regression of Figure 3 and can be described through Equation (3).

(3) $$\Delta {\mathrm{CO}}_{2}=-\frac{\ln\left(\frac{vol-4.677}{66.154}\right)}{0.207}$$

where *vol* corresponds to the available volume per occupant and ΔCO_2_ to the increase in CO_2_ concentration inside the room. The r-squared measure of determination of the non-linear regression shown in the image is 0.60. This value can be explained by the fact that on windy days, the increase in CO_2_ is lower, which shows the existence of significant air filtrations in classrooms.

From Equation (3), it was calculated that maintaining a volume per occupant of 7.46 m^3^, which corresponds to an occupancy of 19 people in a 140 m^3^ classroom, 716 ppm would be reached in 19 min, 816 ppm in the following 6 min, and 1016 ppm in a further 13 min. However, when the volume per occupant increases to 16.03 m^3^, corresponding to an occupancy of 9 in this size of room, the times change to 35 min, 12 min, and 23 min, respectively.

**Figure 4:**
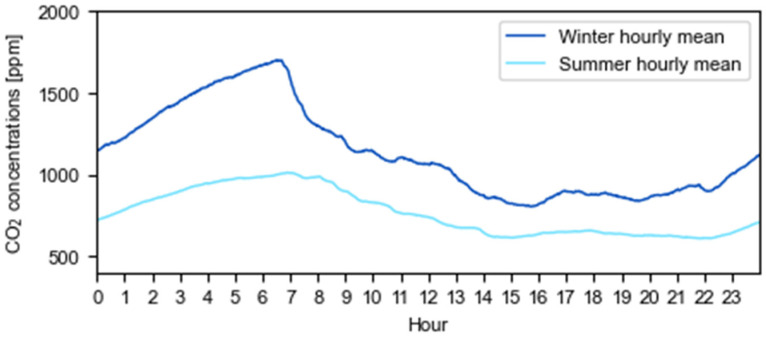
Daily average evolution of CO_2_ concentrations in homes for winter and summer seasons.

In homes, the average daily CO_2_ pattern at study places (Figure 4) shows that there is no ventilation at night when CO_2_ levels ascend at a rate of 90 ppm/h or 1.5 ppm/min. After 3 h without ventilation, CO_2_ levels surpass 1200 ppm. Consequently, data from 00:00 h to 6:58 h is filtered from the sample as it is considered that the room is used for sleeping. Median values remain below 900 ppm during the winter and below 700 ppm in the summer. As summarized in Table 4, during the cold season, 80% of the time the CO_2_ levels remain below 1216.45 ppm, which corresponds to IAQ_II_ or better, and in the warm season this value reaches 89%. Additionally, in the winter, 91% of the concentrations do not exceed 1766.45 ppm (IAQ_III_) while in the summer the percentage is 4 points higher. These measured values are considered acceptable in relation to the studied building type.

**Figure 5:**
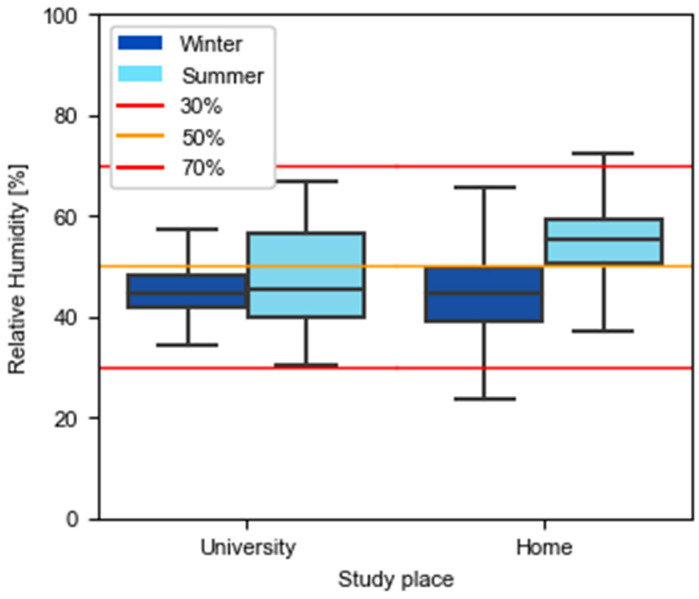
Recorded relative humidity in universities and homes during winter and summer seasons.

**Figure 6:**
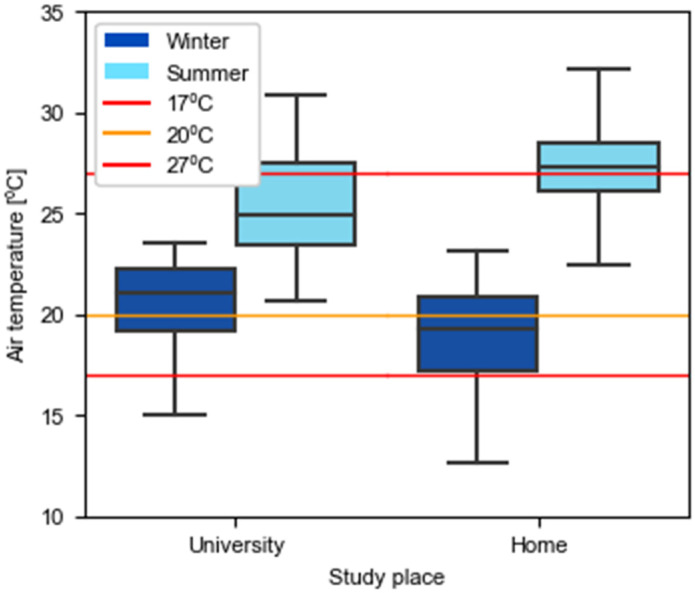
Recorded air temperature in universities and homes during winter and summer seasons.

To make Figure 5 and Figure 6 more intuitive, a color code has been established where red corresponds to environments that do not comply with the limits indicated in RD 486/1997 [33], orange when the temperature is between 17 and 20 °C and the relative humidity between 30 and 50%, and green for cases in which the ranges of 20–27 °C and 50–70% of relative humidity are satisfied.

Relative humidity values exhibit similar levels in homes and universities, mostly in the winter. Homes have a 10% difference in the summer median compared to the winter median. This can be explained since the average external relative humidity during the winter measurements period was 46%, while in summer the mean relative humidity was 60%.

In general, the thresholds of 30% and 70% were followed. Only 4% of the overall measurements carried out in homes were outside this range (Table 4) but may have been derived from activities that are unrelated to the study.

Figure 6 reveals that homes reach lower temperatures during the winter and higher values during the summer. The summer median temperature at home is above 27 °C whereas universities only reach 25 °C. The main reason for this is that the homes’ mean *T*_*o*,*rm*_ of the summer measurements is 4 °C higher than that of universities, since the measures were mostly taken before the end of May, when classes finish, while household measures were mainly made during the exam period in June. Additionally, a heatwave hit Catalonia in June [42], which may explain the high temperatures recorded during that period. In winter, the mean *T*_*o*,*rm*_ were more similar: 11.3 °C during the university measurement period and 12.6 °C during the home measurements. However, when the indoor environment was analyzed, lower temperatures were measured at homes, which could be due to the more restrained use of heating systems. In the case of dwellings, the times at which the room might be unoccupied were analyzed.

In terms of the acceptable temperature range set in RD 486/1997 [33], the regulation was met more frequently during the winter with a percentage of time below 17 °C of 7% in universities and 22% in houses, than during the summer, with periods above 27 °C of 29% in universities and 58% in houses.

**Figure 7:**
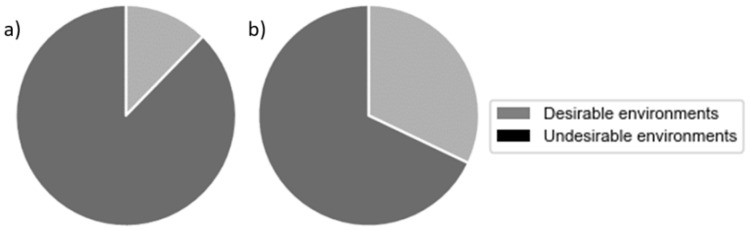
Percentage of time in a desirable and undesirable environment for (**a**) winter and (**b**) summer seasons at university.

In an analysis of the recommended temperature and relative humidity to minimize influenza transmission, at universities, 35% of the recordings showed temperatures lower than 20 °C. In addition, 79% of the time in the winter season and 67% in the summer season the relative humidity did not reach 50%. Considering that, to reduce contagion, recommendations indicate that both parameters must exceed the set thresholds (20 °C and 50%) at the same time, it was concluded that during the winter university spaces were undesirable environments 88% of the time (Figure 7). During the summer, this issue had less impact as the temperatures were always above 20 °C. However, it was still critical up to 68%.

**Figure 8:**
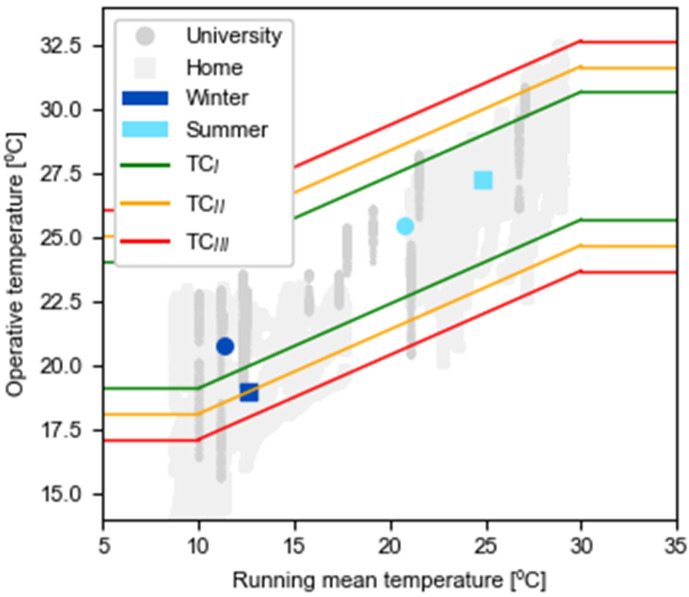
Operative temperature at university and homes according to comfort categories.

Figure 8 shows the results of the application of the adaptive thermal comfort model for the performed measures. The results show that the mean values for both study spaces were within the limits. Despite the average values, there were situations in which the operative temperature fell outside the limits. Consequently, in dwellings during the winter, 33% of the time, this parameter was below the lower limit set in the category TC_IV_.

**Figure 9:**
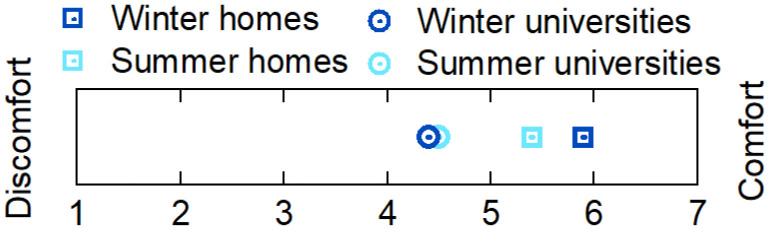
Thermal comfort perception at university and homes.

The results of the university and home point-in-time surveys are shown in Figure 9. At universities, responses were obtained in each of the measurements with an average participation of 84% while at homes, ten out of thirteen participants (77%) answered at least one daily environmental comfort survey.

The perceived thermal comfort was rated as lower at university than at home during both seasons. This evaluation disagrees with the results obtained by the mathematical model from the analysis of data collected during the winter season. Moreover, at universities the comfort during the summer was slightly better than during the winter, at 4.5 vs. 4.4. The results indicate the opposite in the case of dwellings, which is understandable since high temperatures spiked during the specific measurement period at home.

The interviews carried out during winter at homes revealed that the thermal comfort was found to be acceptable (5.9/7, where 7 is comfortable). Nonetheless, this score dropped to 4.4 when participants were asked about thermal comfort in the household characterization survey. A similar result was found for summer. The thermal comfort was acceptable (5.4/7) with a slightly warm perception (0.9) when the point-in-time surveys were analyzed. However, it dropped to 3.7 when the characterization survey was analyzed.

Lastly, a total of 43 users of the university installations answered the general survey, 21% of whom only answered the question on their perception about the thermal comfort in winter for both working spaces, university and home. The rest of the respondents fully completed the questionnaire. Among them, 85% indicated that they had a specific space to study or work in their homes. In terms of their preferred study place, 65% preferred to study from home, 15% from university, and the remaining 20% were indifferent. The first group indicated that they preferred studying at home since they could choose the temperature (59%) and because the classrooms were naturally ventilated (27%). Among the comments collected from this group, reference was made mainly to a lack of adequate air-conditioning facilities at university, which makes it difficult to concentrate, and one respondent also indicated a lack of power sockets, which resulted in overcrowded versus unoccupied university spaces. Regarding the participants who would rather study at university, one participant felt worse in terms of indoor environmental quality at university than at home but preferred face-to-face education, as online education made the process lonelier. Another participant indicated feeling fine in both spaces in terms of thermal comfort, and another indicated that temperatures were inadequate at home. As for the participants who did not prefer one space over another, three pointed to natural ventilation as a source of discomfort in universities, and four reported suffering from inadequate temperatures at home.

**Figure 10:**
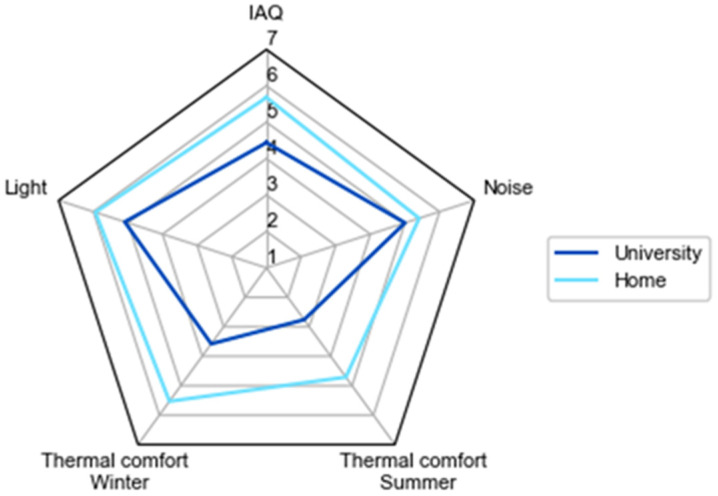
Assessment of indoor environmental quality at university and home by means of the IAQ, Light, Noise and Thermal Comfort in the winter and the summer.

Figure 10 summarizes the respondents’ overall perceptions about the indoor environmental parameters evaluated through the survey. The large difference in comfort described in each space is notable. The perception of the environment at the university was much poorer, especially in terms of thermal comfort in summer.

## 4. Discussion

First, it should be mentioned that all the studied university buildings were built before 1980, which means that no energy efficiency regulations for the construction of new buildings had been approved. The energy inefficiency is reflected in the energy certifications and, more specifically, in the section of heating demand, which in all three cases obtained a G classification, the worst rating on the scale used in the certifications. Additionally, the availability of refrigeration systems in these buildings is almost nonexistent.

In the case of the residential buildings, 73% were built prior to the regulation approval, which is not surprising since 69% of the constructions in Barcelona are older than the energy efficiency law [43]. In terms of heating systems, 73% of the households have a specific heating system installation (water radiators or heat pump), and only 23% of them have electric radiators or no system at all. These results are presented in contrast to the results of the PROJECT SECH-SPAHOUSEC, which indicates that in the Mediterranean area, the main energy source for heating is predominantly electrical [44]. This difference could be derived from the small group of dwellings analyzed in the study and from the economic profile of the students attending the analyzed universities. In terms of refrigeration systems, participant households are better equipped than university buildings, with 45% of the dwellings having cooling systems and 27% of them having fans.

The analysis of the CO_2_ concentration shows lower levels at university than in homes, even when night-time is filtered from the sample. However, if acceptable quality ranges are considered, the results end up being worse at universities than in homes, as IAQ_III_ is valid for houses but not for university classrooms. Adding the percentage of time at IAQ_III_ and IAQ_IV_ from Table 4, the risk of SARS-CoV-2 at universities is considered high or very high 21% of the time in the winter. This value is lower during the summer (7%) since occupants tend to open more windows, which results in better ventilation rates. These situations could be mitigated if measures were taken to reduce classroom occupancy levels. This was the intention when the UPC indicated that 70% was the capacity limit. However, this limit was not enough to keep CO_2_ levels under control with minimal disruption in the classrooms. For example, considering a 140 m^3^ classroom with 30 seating places, this occupancy level corresponds to a volume per occupant of 6.7 m^3^, increasing CO_2_ concentration by 16.93 ppm/min. Therefore, keeping a maximum concentration of 816 ppm would mean opening windows approximately every 20 min, while reducing the occupancy to 50% or 20% would require ventilation every 30 min or 60 min, respectively. Given these circumstances and when facing cold seasons, a balance between low-risk and thermally comfortable environments should be maintained, since reducing occupancy to 20% seems impossible due to a lack of resources in terms of space and professors.

In this regard, Equation (3) could be used to determine the optimal occupancy levels depending on the characteristics of the classroom. In addition, this tool can also be used in combination with mixed-mode ventilation strategies to reduce energy consumption. This is consistent with studies that concluded that this type of ventilation is an effective way to achieve energy savings in the fight against climate change when considering mild seasons and temperate regions [45,46,47]. However, to implement the proposed tool in management systems, the value of the r-squared of the non-linear regression (0.6) should be improved by gathering more data.

Regarding relative humidity, the legal thresholds established for sedentary jobs are being followed. As for the temperature and comfort thresholds, universities seem to have longer time periods following the legal temperature ranges and ensuring the acceptable comfort limits set in ISO EN16798–1–2019. Nevertheless, if the conditions for reducing the influenza contagion risk are evaluated, it should be mentioned that more than half the time these requisites are not met.

The results of the point-in-time surveys are in disagreement with the measured data. Thermal comfort is rated better in houses than in universities with differences in ratings of 1.5 in winter and 0.9 in summer. This discrepancy could be related to the fact that thermal adaptability is higher in households. Interestingly, perceptions of thermal comfort are lower in the household characterization survey and in the general survey. These results show that people tend to give a more negative rating when they are asked about their thermal comfort for long timespans than when they are asked about their present state. The rating could be up to 24% worse, which could be understood by the fact that, when asked over a longer period, the response can be influenced by external factors such as health or mood among others. In any case, the results of the general survey should be highlighted, since they show that the environment is better rated at home than at universities with a big difference in thermal comfort.

From these analyses, it seems that online education should be the answer to health emergencies only. However, when looking at the suitability of spaces for performing educational tasks, it should be noted that during both winter and summer, higher CO_2_ levels have been recorded in dwellings, and, even if the examined households do not reach the thresholds provided by some researchers for experiencing a drop of certain cognitive abilities [23,24], their CO_2_ concentrations are not too far from them. In addition, the use of other heating systems could easily surpass those limits, such as the use of butane heaters according to a study conducted in Barcelona [48]. At home temperatures also show more extreme values, both high and low, which, apart from affecting performance [49,50], may also demotivate students [26].

Nonetheless, the academic outcomes of students enrolled in courses related to project development, management, and leadership offered at the ETSEIB and the ESEIAAT (which coincide with the courses and schools monitored in this study) indicate that no impact on the academic performance of students was perceived during the lockdown [51]. Although this might not be the case in this study, it should be highlighted that uneven access to technology may have deepened learning inequalities during the pandemic [52].

This study is not exempt from limitations. First of all, the results of the study are limited by the quantity of conducted measurements and by the number of participants; therefore, enlarging the sample by performing more measurements during longer periods and at different locations, as well as reaching a bigger group of students, would strengthen the outcome of the study. This would allow further classification of the data (not just winter and summer seasons), and the results may reveal some changes in patterns. Additionally, due to the academic calendar, the university monitoring periods do not coincide with those at home, so the difference in weather conditions may have favored the universities’ temperature results.

## 5. Conclusions

This study characterizes on-site and online education from an environmental quality perspective to verify whether current conditions are adequate for pandemic situations and from the perspective of basic learning conditions.

Considering the preferences of students and the results of the analysis, it can be concluded that universities do not have adequate facilities, especially during pandemic periods when environmental conditions require greater control. However, adequate management of the occupation rates of spaces would result in safer environments and clearer ventilation instructions. This seems a better solution than shifting to online education since it could reduce students’ performance and motivation while deepening social inequalities.

This study presents initial trends with a reduced number of measurements. Therefore, to statistically reinforce these trends, future work should enlarge the sample and increase student participation. Additionally, it should be highlighted that the results cannot be applied globally as educational environments and climatic circumstances might be different in other territories.

## Figures and Tables

**Table 1:** Sensor technical specifications.

	Sensor	Measuring Range	Resolution	Accuracy
Air temp. (°C)	Comet	−20–60	0.1	±0.4
	DeltaOHM	−20–80		±0.1 (20–60)/±0.2 (rest)
CO_2_ concentrations (ppm)	Comet	0–5000	1	±50 + 3% from reading
	DeltaOHM			
Relative Humidity (%)	Comet	0–100	0.1	±1.8
	DeltaOHM			±2 (0–80)/±3 (rest)

**Table 2:** CO_2_ concentration quality code by ranges for university and home workplaces.

Code	University	Home
IAQ_I_	<716 ppm	<966 ppm
IAQ_II_	716 ppm–816 ppm	966 ppm–1216 ppm
IAQ_III_	816 ppm–1016 ppm	1216 ppm–1766 ppm
IAQ_IV_	>1016 ppm	>1766 ppm

**Table 3:** Temperature ranges according to the adaptive thermal comfort model.

Code	Rate	Indoor Environment
TC_I_	High comfort	*T_op_* = 0.33·*T*_*o*,*rm*_ + 18.8 + 2
		*T_op_* = 0.33·*T*_*o*,*rm*_ + 18.8 − 3
TC_II_	Medium comfort	*T_op_* = 0.33·*T*_*o*,*rm*_ + 18.8 + 3
		*T_op_* = 0.33·*T*_*o*,*rm*_ + 18.8 − 4
TC_III_	Moderate comfort	*T_op_* = 0.33·*T*_*o*,*rm*_ + 18.8 + 4
		*T_op_* = 0.33·*T*_*o*,*rm*_ + 18.8 − 5
TC_IV_	Low comfort	

**Table 4:** Percentage of time per category for the parameters: CO_2_ concentration, temperature, relative humidity and thermal comfort.

Ranges	University		Home	
	Winter	Summer	Winter	Summer
CO_2_ Concentrations				
IAQ_I_	66%	89%	67%	82%
IAQ_II_	13%	4%	13%	7%
IAQ_III_	10%	6%	11%	6%
IAQ_IV_	11%	1%	9%	5%
Temperature				
<17	7%	-	22%	-
17–20	28%	-	37%	-
20–27	65%	71%	41%	42%
>27	-	29%	-	58%
Relative Humidity				
<30%	-	-	3%	1%
30%–50%	79%	68%	72%	22%
50%–70%	21%	32%	25%	77%
>70%	-	-	-	-
Thermal Comfort				
TC_I_	67%	78%	29%	96%
TC_II_	15%	16%	22%	3%
TC_III_	4%	3%	16%	1%
TC_IV_	14%	3%	33%	-

## Data Availability

The data are not publicly available for confidential reasons.
